# 

**Published:** 1908-06

**Authors:** 


### CONTENTS.

ORIGINAL COMMUNICATIONS—
Medical Organization. By M. A. Clark, M. D., Macon Ga. .. .. .. .. .. .. .. .. .. .. .. 123
A Report on One Hundred and Twenty Knee Joint Operations. By Michael Hoke, M. D., and C. R. Andrews, M. D., Atlanta, Ga. .. .. .. .. .. .. .. .. .. .. .. .. .. .. .. .. 132
Tumors of the Bladder. By Alfred L. Fowler, M. D., Atlanta, Ga. .. .. .. .. .. .. .. 146
"American Hookworm"—Uncinariasis. Deductions drawn from treatment of 408 cases. By A. G. Fort, Ph. B., M. D., Lumpkin, Ga. .. .. .. .. .. .. .. .. .. .. .. .. .. 154
A Case of Scrotal Haematocele. By Whatley W. Battey, Jr., M. D., Assistant Professor, Anatomy and Clinical Surgery, Medical Department, University of Georgia. .. .. .. .. 158
Prophylaxis and Treatment of Typhoid Fever. By John B. Woodville, M. D., Fayette, W. Va., 161
EDITORIALS—
The Antivivisectionist. .. .. .. .. .. .. .. .. .. .. .. .. .. .. .. .. .. .. .. 167
The Crawford W. Long Monument .. .. .. .. .. .. .. .. .. .. .. .. .. .. .. .. 168
Commencement of the Atlanta School of Medicine .. .. .. .. .. .. .. .. .. .. .. 169
Commencement Exercises of the Atlanta College of Physicians and Surgeons .. .. .. .. 170
Object to Automobile Law .. .. .. .. .. .. .. .. .. .. .. .. .. .. .. .. .. .. 171
Dr. Knopf on Consumption .. .. .. .. .. .. .. .. .. .. .. .. .. .. .. .. .. .. 172
Annual Report of the Bureau of Health of the Philippine Islands .. .. .. .. .. .. .. 172
Death of Dr. Wimberly .. .. .. .. .. .. .. .. .. .. .. .. .. .. .. .. .. .. .. 174
BOOK REVIEWS.. .. .. .. .. .. .. .. .. .. .. .. .. .. .. .. .. .. .. .. .. .. 174
NEWS AND NOTES .. .. .. .. .. .. .. .. .. .. .. .. .. .. .. .. .. .. .. .. .. 178

